# Safety, tolerability, pharmacokinetics, and immunogenicity of an anti-SARS-CoV-2 monoclonal antibody HFB30132A after single dose intravenous administration in healthy Chinese subjects: a phase 1, randomized, double-blind, placebo-controlled study

**DOI:** 10.3389/fphar.2023.1117293

**Published:** 2023-05-23

**Authors:** Shanshan Li, Xiaojie Wu, Nanyang Li, Guoying Cao, Jingjing Wang, Yuancheng Chen, Size Li, Jinjie He, Jufang Wu, Haijing Yang, Ke Lin, Chao Qiu, Angela Liu, He Zhou, Francisco Adrian, Liang Schweizer, Wenhong Zhang, Jingwen Gu, Jing Zhang

**Affiliations:** ^1^ Huashan Worldwide Medical Center, Huashan Hospital, Fudan University, Shanghai, China; ^2^ Phase 1 Clinical Research Center, Huashan Hospital, Fudan University, Shanghai, China; ^3^ Department of Infectious Diseases, Huashan Hospital, Fudan University, Shanghai, China; ^4^ HiFiBiO (Hang Zhou) Ltd., Shanghai, China; ^5^ Institute of Antibiotics, Huashan Hospital, Fudan University, Shanghai, China

**Keywords:** neutralizing antibody, monoclonal antibody, SARS-CoV-2, HFB30132A, COVID-19, safety, pharmacokinetics, immunogenicity

## Abstract

**Objective:** The pandemic of coronavirus disease 2019 (COVID-19) caused by severe acute respiratory syndrome coronavirus-2 (SARS-CoV-2) still protracts worldwide. HFB30132A is an anti- SARS-CoV-2 monoclonal antibody purposely engineered for an extended half-life with neutralizing activity against majority of the virus variants identified so far. The aim of this study was to evaluate the safety, tolerability, pharmacokinetics (PK), and immunogenicity of HFB30132A in healthy Chinese subjects.

**Methods:** A phase 1, randomized, double-blind, placebo-controlled, single ascending dose clinical trial was designed. Twenty subjects were enrolled to Cohort 1 (1,000 mg dose level, 10 subjects) or Cohort 2 (2,000 mg dose level, 10 subjects). Subjects in each cohort were assigned randomly to receive a single intravenous (IV) dose of HFB30132A or placebo at a ratio of 8:2. Safety was assessed in terms of treatment emergent adverse events (TEAEs), vital signs, physical examination, laboratory tests, and ECG findings. PK parameters were measured and calculated appropriately. Anti-drug antibody (ADA) test was performed to detect anti-HFB30132A antibodies.

**Results:** All subjects completed the study. Overall, 13 (65%) of the 20 subjects experienced TEAEs. The most common TEAEs were laboratory abnormalities (12 subjects [60%]), gastrointestinal disorders (6 subjects [30%]), and dizziness (4 subjects [20%]). All TEAEs were Grade 1 or Grade 2 in severity based on the criteria of Common Terminology Criteria for Adverse Events (CTCAE). Serum exposure (C_max_, AUC_0-t,_ AUC_0-∞_) of HFB30132A increased with ascending dose. After single dose of 1,000 mg and 2000 mg HFB30132A, the mean C_max_ was 570.18 μg/mL and 898.65 μg/mL, the mean AUC_0-t_ value was 644,749.42 h*μg/mL and 1,046,209.06 h*μg/mL, and the mean AUC_0-∞_ value was 806,127.47 h*μg/mL and 1,299,190.74 h*μg/mL, respectively. HFB30132A showed low clearance ranging from 1.38 to 1.59 mL/h, and a long terminal elimination half-life (t_½_) of 89–107 days. ADA test did not detect any anti-HFB30132A antibodies

**Conclusion:** HFB30132A was safe and generally well-tolerated after single IV dose of 1,000 mg or 2000 mg in healthy Chinese adults. HFB30132A did not induce immunogenic response in this study. Our data support further clinical development of HFB30132A.

**Clinical Trial Registration:** https://clinicaltrials.gov, identifier: NCT05275660.

## 1 Introduction

According to the World Health Organization (WHO) COVID-19 dashboard, there have been more than 520 million cumulative confirmed cases and over 6 million deaths globally as of 18 May 2022 (https://www.who.int/). The pandemic of COVID-19 still protracts worldwide. More public health efforts are required to contain the epidemic due to the emerging SARS-CoV-2 variants, such as Delta and Omicron. Vaccination is considered the first priority for all eligible individuals to prevent COVID-19-related severe diseases. However, for the purpose to effectively protect those who are moderately or severely immunocompromised and have an inadequate immune response to COVID-19 vaccination and those who are not able to be fully vaccinated with any available COVID-19 vaccines due to a history of severe adverse reactions to a COVID-19 vaccine or any of its components, the U.S. Food and Drug Administration (FDA) recently issued an Emergency Use Authorization (EUA) for tixagevimab and cilgavimab, a combination of two long acting anti-SARS-CoV-2 monoclonal antibodies (mAbs), as an alternative pre-exposure prophylaxis (National Institutes of Health, 2022). Multiple anti-SARS-CoV-2 mAbs, such as bamlanivimab plus etesevimab (Dougan et al., 2022), casirivimab plus imdevimab (RECOVERY Collaborative Group Abbas et al., 2022), and sotrovimab (Gupta et al., 2022), bebtelovimab (Westendorf et al., 2022) have shown promising results in lowering viral load, relieving symptoms, and avoiding hospitalization in patients with mild or moderate COVID-19 who are at high risk for progression to severe disease or hospitalization. In addition, a single intramuscular dose of tixagevimab plus cilgavimab showed statistically and clinically significant protection against progression to severe COVID-19 or death *versus* unvaccinated individuals with placebo, associated with good tolerability and safety in TACKLE trial, a phase 3, randomized, double-blind, placebo-controlled study conducted in the USA, Latin America, Europe, and Japan (Montgomery et al., 2022).

HFB30132A is a fully human anti-SARS-CoV-2 mAb targeting spike (S) protein receptor binding domain (RBD). It was identified from a convalescent COVID-19 patient and purposely engineered as an immunoglobulin (Ig) G4 subclass with 4 mutations: the S228P mutation to prevent half antibody formation and the M252Y/S254T/T256E (YTE) mutation to enable a longer half-life (Dall'Acqua et al., 2006; Robbie et al., 2013). IgG4 antibodies with YTE mutation have shown increased binding to FcRn, which enables longer half-life, and reduced binding to crystallizable fragment γ receptors (FcγR), which could reduce or eliminate the risk of antibody dependent enhancement (ADE) effect (Liu et al., 2019; Graham, 2020; Guo et al., 2021), and may increase distribution of the antibodies to the respiratory system mucosa compared to IgG1 antibodies (Dall'Acqua et al., 2006; Tabrizi et al., 2019; Spiess et al., 2013; Ko et al., 2014).

The aim of this study was to evaluate the safety, tolerability, pharmacokinetics (PK), and immunogenicity of HFB30132A after single dose intravenous (IV) infusion in healthy Chinese adults and provide essential data for further clinical development of HFB30132A. We have also considered the possible role of variants of concern (VOCs) when designing HFB30132A. The VOCs included B.1.1.7, B.1.617.2, C.37, B.1.617.1, B.1.526, and B.1.621 variants. The *in vitro* experiment has been conducted to determine the potential neutralization activities of HFB30132A against these VOCs and the results will be available later.

## 2 Methods

### 2.1 Ethics statement

The study protocol and informed consent form (ICF) were approved by Huashan Hospital Institutional Review Board [No. 2021 (020)]. The rationale, procedures, and objectives of the study and the potential risks and benefits were explained to each subject in details. Each subject was informed of his/her right to withdraw from the study at any time for any reason. All subjects signed and dated the approved ICF prior to initiation of any study procedures. The study was conducted in compliance with the principles of the Declaration of Helsinki and the International Conference on Harmonization Good Clinical Practice guidelines. This clinical study is registered at ClinicalTrials.gov (identifier: NCT05275660).

### 2.2 Dose selection

A single IV dose of 10 mg/kg HFB30132A (infusion over 60 min) in non-human primates (NHP) resulted in a combined (males and females) mean area under the serum drug concentration-time curve up to 1,345 h (8 weeks) after start of treatment (AUC_0-1345_) of 77,500,000 ng × hour/mL and mean maximum observed serum concentration (C_max_) of 266,000 ng/mL. The mean time to C_max_ (T_max_) was 1.0833 h and the mean terminal half-life (t½) was 459 h after start of treatment. No adverse clinical observations or body weight changes were recorded from Day 1 through Day 56 of the study.

In the 2 weeks repeat dose NHP toxicity study of HFB30132A, no treatment-related adverse events were observed through Day 15 (dosing phase) or Day 71 (recovery phase) after 2 weekly administered IV doses of 300 mg/kg or below.

Initial dose design: In the repeat dose toxicity study of HFB30132A in NHP, the no observed adverse effect level was 300 mg/kg. HFB30132A is a macromolecular biological product (molecular weight >100 kDa). It mainly distributed in plasma. When calculating the human equivalent dose, the body surface area is generally not converted. Hence, the safety factor is set to 10, so 30 mg/kg should be safe in human body. In addition, the dose of 1,000 mg is the intermediate dose in the phase I clinical trial currently conducted in the United States, and the safety was supported by the data of the first-in-human clinical trial (data unpublished). Therefore, 1,000 mg was selected as the initial dose in this clinical study.

Maximum dose design: The estimated maximum dose is generally below 1/5–1/2 of the maximum tolerated dose in animals based on the Dollery method. The maximum tolerated dose of HFB30132A in NHP is 300 mg/kg, and the recommended maximum dose in humans can be 60–150 mg/kg. According to the efficacy study of HFB30132A in rhesus monkeys COVID-19 model, 10 mg/kg and 50 mg/kg can produce therapeutic effects. Therefore, 2,000 mg would be appropriate as the maximum dose group in this clinical study. If the maximum dose was reached, but the standard for terminating dose escalation was not met, the sponsor would decide whether to continue dose escalation after considering the recommendations of the Independent Data Monitoring Committee (IDMC).

### 2.3 Study design and dose escalation

The study was a randomized, double-blind, placebo-controlled, single ascending dose, phase 1 study conducted to evaluate the safety, tolerability, PK, and immunogenicity of HFB30132A after single dose IV infusion in healthy Chinese adults.

A total of 20 subjects were enrolled to Cohort 1 (1,000 mg dose level, 10 subjects) or Cohort 2 (2,000 mg dose level, 10 subjects). There were at least 3 males or females in each cohort. The first 5 subjects in Cohort 1 were randomized to receive either HFB30132A or placebo at a ratio of 4:1. The subjects were monitored in the study site for 24 h after dosing for evaluation of the safety and tolerability. If any safety concern was raised by the DEC, or any of the following dose stopping rule was met, the dose escalation would be stopped temporarily, and the events were reviewed immediately. The DEC would evaluate the relationship between the event and HFB30132A dosing in an unblinded manner and review all of the data available to decide whether to continue dose escalation or not.

The dose escalation from a previous cohort to the next cohort would be stopped if one or more of the following stopping criteria were met:

Two or more subjects developed a Grade 3 AE, study drug-related or probably related in the opinion of the investigator or sponsor, except infusion reaction (local or systemic) that can be managed by symptomatic treatments, e.g., non-steroidal anti-inflammatory drugs or anti-histamines; Any Grade 4 or greater event in any single subject that resulted in discontinuation of further dosing, or unblinding of study treatment for the affected subject(s); If an event corresponding to the above criteria was deemed study drug-related or probably related and occurred in the previous cohort after the 7-day observation period, further progression to next dose group or cohort would be stopped temporarily and the DEC would review the data available and decide whether to continue the study or not.

The DEC would recommend dose modification based on safety and PK data review for the cohorts following Cohort 1.

If no subject met the criteria for terminating dose escalation, the remaining 5 subjects in the cohort were randomized to receive HFB30132A or placebo. Dose escalation was terminated when half number of the subjects developed AEs of Common Terminology Criteria for Adverse Events (CTCAE) Grade 2 or higher; 2 or more subjects developed AEs of CTCAE Grade 3; 1 or more subjects developed AEs of CTCAE Grade 4 or higher. Dose escalation proceeded from 1,000 mg to 2000 mg based on the safety assessment of the study drug 14 days following initial dose administration. All of the 10 subjects in the 2,000 mg cohort were dosed on the same day.

### 2.4 Randomization and blinding

This study was double-blind until the end of the study. The randomization assignment was not revealed to study subjects, Investigator, study site personnel (except for designated unblinded staff who handled the study drug and predefined unblinded teams of the Sponsor and CRO) or the sponsor and its representatives until all the final clinical data were entered into the database, and the database was locked and released for analysis. The designated nurses at the study site, who were blinded to the treatment assignment were responsible for preparing and dispensing the study medication. All study drugs were delivered to the study site and were assigned to treatment groups by the pharmacy personnel in accordance with the provided randomization schedule.

A randomization list was generated using SAS Ver 9.4 to randomly assign subjects to receive HFB30132A or placebo at the trial center. A total number of 20 subjects were enrolled. Specifically, 10 subjects were assigned to 1,000 mg cohort and another 10 subjects to 2,000 mg cohort, at least 3 males or females in each cohort. The subjects in each cohort were randomized to receive either HFB30132A or placebo at a ratio of 4:1. The study drug preparation was masked by a coded identification label. The matching placebo had the same ingredients as HFB30132A formulation but did not contain the mAb. The placebo was indistinguishable from HFB30132A formulation. The investigators, study nurses, study staff, the sponsor, and the healthy volunteers were blinded to the treatment assigned to each subject.

### 2.5 Participants and drug administration

An eligible subject should be a healthy Chinese male or a non-pregnant, non-lactating female, aged 18–60 years (inclusive). Male subjects should have a body weight ≥50 kg, and females should have a body weight ≥45 kg. Healthy subjects implied no clinically significant abnormality based on a thorough evaluation by doctors, including medical history, full physical examination, vital signs, electrocardiogram (ECG), and clinical laboratory tests prior to administration of the study drug. Each subject received single dose IV administration of HFB30132A or matching placebo over 90 min (±15 min) on Day 1.

### 2.6 Safety assessment

Safety assessment was performed throughout the entire study of 270 days. All subjects received ECG monitoring within 2.5 h (±15 min) after IV infusion. Vital signs, physical examination, laboratory tests, treatment emergent adverse events (TEAEs), and concomitant medications were monitored on days 1, 2, 7, 30, 60, 150, 270 or at early discontinuation. All TEAEs were collected and assessed by physicians. Including adverse drug reactions (ADRs), treatment-emergent serious AEs (TESAEs), treatment-emergent AEs of special interest (TEAESIs), and emerging clinical conditions that occurred within 270 days after HFB30132A infusion. CTCAE 5.0 grading scale was used for AE reporting and severity assessment. ADRs referred to any untoward medical occurrence in a subject after administration of a study drug. TEAESIs included infusion related reactions such as hypersensitivity, anaphylactic reactions, and local intolerability.

### 2.7 PK and immunogenicity sample collection and assessment

Individual PK samples were collected over 270 days at the following time points and acceptable tolerance windows: prior to study drug administration on day 1 (any time), and then at end of infusion (±2 min), 120 min (±5 min), 2.5 h (±5 min), 5.5 h (±5 min), 9.5 h (±5 min), and 13.5 h (±5 min) after start of study drug infusion, and at 24 and 48 h post-dose on day 2 and day 3; and on days 7, 14, 30, 45, 60, 90, 150, 210, and 270 post-dose, or at the time of early discontinuation. Immunogenicity samples were collected prior to study drug administration on day 1 (any time), and on days 7, 30, 60, 150, and 270 post-dose; or at the time of early discontinuation. The serum concentrations of HFB30132A were determined using a validated enzyme-linked immunosorbent assay (ELISA), the parameters of which are shown in Table 1. Anti-HFB30132A antibodies in serum were detected on the Meso Scale Discovery (MSD) platform using a validated electrochemiluminescence immunoassay–affinity capture elution bridging method. This method includes a tiered approach, i.e., first define positivity (tier 1), then the specificity of the positivity assay (tier 2), and finally to titer the responses of samples confirmed to be specific (tier 3).

**TABLE 1:** Parameters of the methods for determination of serum concentration of HFB30132A.

Validation parameter	Results	Acceptance criteria
Method	ELISA	
LLOQ		15.6 ng/mL
ULOQ		2000.0 ng/mL
Accuracy and precision	ULOQ & LLOQ	(1) Intra- and Inter-batch ULOQ & LLOQ
	Intra-batch accuracy RE%	Accuracy: RE% within ±25%
	−10.7% to −0.6%	Precision: CV% ≤ 25%
	Intra-batch precision CV%	QC: RE% within ±20%
	1.4%–8.4%	Accuracy: RE% within ±20%
	Inter-batch accuracy RE%	Precision: CV% ≤20%
	−6.6% to −6.4%	
	Inter-batch precision CV%	
	5.5%–6.2%	(2) Total error between batches
	Total error between batches	ULOQ & LLOQ: 40%
	11.2%–12.8%	QC: 30%
	QC	
	Intra-batch accuracy RE%	
	−13.7%–0.9%	
	Intra-batch precision CV%	
	0.2%–10.0%	
	Inter-batch accuracy RE%	
	−6.8% to −4.6%	
	Inter-batch precision CV%	
	4.4%–7.2%	
	Total error between batches	
	11.2%–12.8%	
Dilution linearity	500-fold, 50-fold, 5-fold and 2-fold dilution	At least 4/5 of the samples for linear verification of each dilution should be within ±20% of the accuracy (RE%). The precision of the samples with the same final concentration and different dilution ratios should be ≤20%
Specificity	Addition of interfering agent 2019-nCoVS protein RBD or anti-JS016 polyclonal antibody did not meet the criteria	(1) The back calculated concentration of blank matrix was BQL
		(2) LLOQ & ULOQ: Re% within ±25%
		(3) If the above criteria cannot be met, the actual results can be reported

ELISA, enzyme-linked immunosorbent assay; LLOQ, lower limit of quantitation; ULOQ, upper limit of quantitation; QC, quality control; CV, coefficient of variation; RE, relative error; RBD, receptor binding domain; BQL, below the quantization limit.

### 2.8 PK analysis

The concentration data of HFB30132A were analyzed to obtain the PK parameters by a non-compartmental analysis method using Phoenix^®^ WinNonlin software (Ver 8.2, Certara, Inc., New Jersey, United States). Actual collection times were used in PK parameter calculations. The following PK parameters were determined: maximum concentration (C_max_), time to reach C_max_ (T_max_), and the area under the serum concentration-time curve from time 0 to the time of the last quantifiable concentration (AUC_0-t_). If the data permitted, elimination rate constant, area under the serum concentration-time curve from time 0 to infinity (AUC_0-∞_), elimination half-life (t_1/2_), clearance (CL), and volume of distribution (V_d_), were further calculated.

### 2.9 Population PK analysis

A PPK model was constructed to characterize the PK profiles of HFB30132A. The base structural model was a three-compartment model with two peripheral compartments and first-order elimination from the central compartment. Interindividual variability (IIV) was evaluated on the volume of central compartment (V1), and volume of the other two peripheral compartments (V2 and V3). Residual errors were described using a proportional error model.

Covariate-parameter relations for evaluation were prespecified based on the decrease of the objective function value (OFV). In the initial modeling, the prespecified covariates included body weight (BW); hematocrit (HCT); hemoglobin (HGB); monocyte (MONO); red blood cell (RBC); age (AGE); diastolic blood pressure (DIABP); respiration (RESP); basophil (BASO); eosinophil (EOS); lymphocyte (LYM); neutrophil (NEUT); white blood cell (WBC); direct bilirubin (BILDIR); bilirubin (BILI); and triglyceride (TG). Finally, the effect of each candidate covariate was investigated by the forward inclusion-backward elimination method. The models were compared in terms of the OFV provided by NONMEM at a significance level of 0.05 (equal to a decrease of 3.84 in the OFV) for inclusion, and 0.01 (equal to an increase of 6.63 in the OFV) for elimination.

The final model was evaluated using nonparametric bootstrap and visual predictive check (VPC) (Bergstrand et al., 2011). The resampling was performed 1,000 times for the bootstrap. The median and the precision of the parameters derived from this analysis were compared with the ones obtained by NONMEM from the whole original data. VPC was performed by simulating 1,000 times from the final model to assess the predictive performance. The PPK model was developed based on first-order conditional estimation with interaction method using Intel Fortran Compiler (GCC, Ver 4.6.0, Free Software Foundation, Inc.), NONMEM (Ver 7.4.1, ICON Development Solutions, Maryland, United States), R (Ver 4.0.2, The R Foundation for Statistical Computing) and Perl‐speaks‐NONMEM (Ver 5.2.6, Uppsala University, Sweden).

### 2.10 Immunogenicity analysis

The immunogenicity of HFB30132A was assessed by an Anti-Drug Antibody (ADA) test to identify the presence or absence of antibodies against HFB30132A. Blood samples were collected on day 1 (−3 to 0 h pre-dose), and days 7, 30, 60, 150, and 270 (the end of the study).

### 2.11 Statistical analysis

The baseline demographic data and safety data of the participants were analyzed using descriptive statistics with SAS^®^ software Ver 9.4. The total sample size of 20 subjects was not based on formal statistical assumptions. This study did not use statistical assumptions as the basis for sample size calculation. Phase 1 studies are usually conducted in healthy volunteers according to FDA’s drug review process for investigational new drugs. This study was designed to investigate the common adverse events and the disposition of the drug in human body. The required number of subjects typically ranges from 20 to 80 (Food and Drug Administration, 2017). Therefore, the number of 10 subjects in each cohort (8 subjects receiving HFB30132A and 2 subjects receiving placebo) was proposed for Phase I study to evaluate the safety, tolerability, PK, and immunogenicity of HFB30132A after intravenous infusion of single ascending doses. The categorical data were summarized with counts and percentages of subjects. The denominator used for the percentage calculation was clearly defined. Continuous data were analyzed with descriptive statistics including where appropriate, n (number of non-missing values), mean, median, standard deviation, minimum, maximum, and coefficient of variation (CV) (%). The immunogenicity of HFB30132A was measured by the prevalence of anti-HFB30132A antibodies. Missing data were not imputed.

## 3 Results

### 3.1 Demographic and other baseline characteristics

Twenty eligible subjects were enrolled and randomly allocated to Cohort 1 (1,000 mg, 10 subjects) or Cohort 2 (2000 mg, 10 subjects). The demographic and baseline characteristics of subjects are presented in Table 2. There were 12 (60%) male subjects (5 each in 1,000 mg group and 2000 mg group, and 2 in placebo group) and 8 (40%) female subjects (3 each in 1,000 mg group and 2,000 mg group, and 2 in placebo group). The mean age of subjects was 32.38 years in HFB30132A 1000 mg group, 26.63 years in 2000 mg group, and 28.25 years old in placebo group. The mean BMI was 23.04 kg/m^2^ in 1,000 mg group, 24.15 kg/m^2^ in 2000 mg group, and 23.88 kg/m^2^ in placebo group. The baseline characteristics were numerically comparable across the dose cohorts. No significant difference was found between HFB30132A groups and placebo group.

**TABLE 2:** Demographic and baseline characteristics of study subjects.

Characteristic	HFB30132A		Placebo	Total
	Cohort 1, 1000 mg (n = 8)	Cohort 2, 2000 mg (n = 8)	(n = 4)	(N = 20)
Age (years)	32.5 (29.0–34.0)	27.0 (23.5–29.5)	27.0 (24.5–32.0)	29.0 (25.0–32.5)
Age range (years)	25–43	22–31	24–35	22–43
Sex, *n* (%)				
Male	5 (62.5)	5 (62.5)	2 (50.0)	12 (60.0)
Female	3 (37.5)	3 (37.5)	2 (50.0)	8 (40.0)
Ethnicity, *n* (%)				
Han Chinese	8 (100)	8 (100)	3 (75.0)	19 (95.0)
Minority Chinese	0 (0)	0 (0)	1 (25.0)	1 (5.0)
Height (cm)	160.60 (156.10–170.90)	167.05 (159.65–168.90)	162.25 (156.10–171.05)	165.45 (155.9–173.9)
Weight (kg)	64.28 (57.70, 68.43)	63.03 (59.30–70.30)	66.60 (54.43–74.30)	64.05 (58.53–70.95)
Body mass index (kg/m^2^)	23.35 (22.35–23.95)	24.95 (21.80–25.60)	24.45 (22.25–25.50)	23.95 (22.10–25.30)
Leukocyte count (10^9^/L)	5.72 (4.67–6.54)	5.96 (5.07–7.54)		
Neutrophil count (10^9^/L)	3.54 (2.84–3.80)	3.66 (3.22–4.44)		
Erythrocyte sedimentation rate (mm/h)	8.00 (4.00–11.00)	4.50 (1.00–11.00)		
Alanine transaminase (U/L)	12.00 (9.00–19.00)	10.00 (8.00–12.00)		
Aspartate transaminase (U/L)	17.50 (13.00–23.00)	14.50 (14.00–19.00)		
Bilirubin (U/L)	6.90 (6.20–8.20)	9.95 (8.10–14.6)		
Creatinine (mg/dL)	79.00 (68.00–88.00)	69.50 (65.00–81.00)		

Data are presented as median (interquartile range) unless otherwise specified.

### 3.2 Safety

**FIGURE 1:**
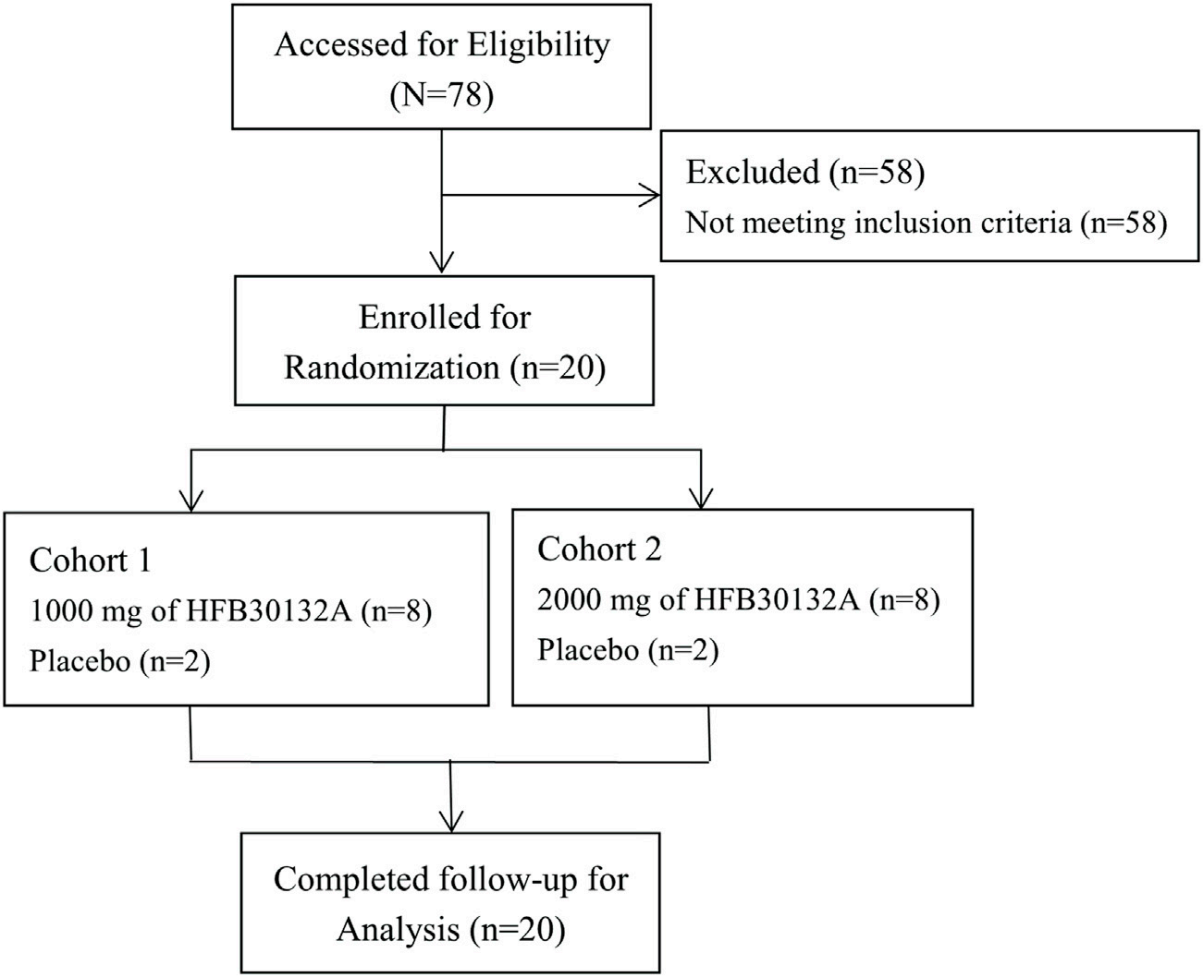
CONSORT flow diagram of study subjects in Phase I clinical trial after intravenous infusion of single dose of 1,000 mg and 2000 mg HFB30132A.

All subjects completed the study and tolerated the study treatment well. No subjects discontinued the study early or discontinued due to COVID-19 (Figure 1). There were no deaths, SAEs, or TEAEs leading to discontinuation throughout the safety assessment up to 270 days after receiving the study drug. Safety assessment was conducted separately for the first 30 days after drug infusion and for the complete 270 days of study. The overall safety summary during the 270 days of study reported that a total of 13 subjects (65.0%) experienced 46 TEAEs: including laboratory abnormalities (60%), gastrointestinal disorders (30%), and dizziness (20%) (Table 3). All TEAEs were rated as CTCAE Grade 1 or 2 in severity. Majority of the TEAEs were laboratory abnormalities, all grade 1 or 2, and resolved soon without any intervention. Among the TEAEs, 5 cases of laboratory abnormalities reported in 4 subjects (20.0%) were considered as ADRs. Specifically, elevated erythrocyte sedimentation rate in 2 subjects (25.0%) in 2,000 mg group and 1 subject (25.0%) in placebo group, and increased neutrophil count and increased leukocyte count in 1 subject (12.5%) in 1,000 mg group. All ADRs were Grade 1 in severity and identified from laboratory tests without symptoms. Majority of the TEAEs were mild in severity and occurred within 30 days post-dose. No specific trends or clinically significant changes were identified in laboratory tests or physical examination findings. No clinically significant changes were observed in vital signs, hypersensitivity triggers, chest X-ray, or 12-lead ECG findings. No allergic reactions (including injection site subcutaneous hemorrhage, pain, swelling, or pruritus) were observed during infusion and post dose.

**TABLE 3:** Summary of HFB30132A safety after single dose intravenous infusion in healthy Chinese subjects.

Safety parameter	HFB30132A				Placebo		Total	
	Cohort 1, 1000 mg (n = 8)		Cohort 2, 2000 mg (n = 8)		(n = 4)		(N = 20)	
	Event, *n*	Subject, *n* (%)	Event, *n*	Subject, *n* (%)	Event, *n*	Subject, n (%)	Event, *n*	Subject, *n* (%)
**TEAEs**	17	5 (62.5)	21	6 (75.0)	8	2 (50.0)	46	13 (65.0)
Laboratory abnormality	9	5 (62.5)	13	5 (62.5)	6	2 (50.0)	28	12 (60.0)
Gastrointestinal disorder	2	2 (25.0)	3	3 (37.5)	1	1 (25.0)	6	6 (30.0)
Infection and infestation	2	1 (12.5)	2	1 (12.5)	1	1 (25.0)	5	3 (15.0)
Upper respiratory tract infection	2	1 (12.5)	1	1 (12.5)	0	0	3	2 (10.0)
Nervous system disorder								
Dizziness	1	1 (12.5)	3	3 (37.5)	0	0	4	4 (20.0)
Skin and subcutaneous tissue disorder								
Eczema	1	1 (12.5)	0	0	0	0	1	1 (5.0)
Mental disorder								
Sleep disorder	1	1 (12.5)	0	0	0	0	1	1 (5.0)
Musculoskeletal disorder	1	1 (12.5)	0	0	0	0	1	1 (5.0)
Joint pain								
**Severity of TEAE**	17	5 (62.5)	21	6 (75.0)	8	2 (50.0)	46	13 (65.0)
Mild (Grade 1)	12	5 (62.5)	17	6 (75.0)	6	2 (50.0)	35	13 (65.0)
Moderate (Grade 2)	5	2 (25.0)	4	2 (25.0)	2	2 (50.0)	11	6 (30.0)
Severe (Grade 3)	0	0	0	0	0	0	0	0
Life threatening (Grade 4)	0	0	0	0	0	0	0	0
**Adverse drug reaction**	2	1 (12.5)	2	2 (25.0)	1	1 (25.0)	5	4 (20.0)
Elevated erythrocyte sedimentation rate	0	0 (0)	2	2 (25.0)	1	1 (25.0)	3	3 (15.0)
Increased leukocyte count	1	1 (12.5)	0	0 (0)	0	0 (0)	1	1 (5.0)
Increased neutrophil count	1	1 (12.5)	0	0 (0)	0	0 (0)	1	1 (5.0)
**Treatment emergent serious AE**	0	0	0	0	0	0	0	0
**TEAE of special interest**	0	0	0	0	0	0	0	0
**Deaths**	0	0	0	0	0	0	0	0

TEAE, treatment emergent adverse event.

### 3.3 Pharmacokinetics

**FIGURE 2:**
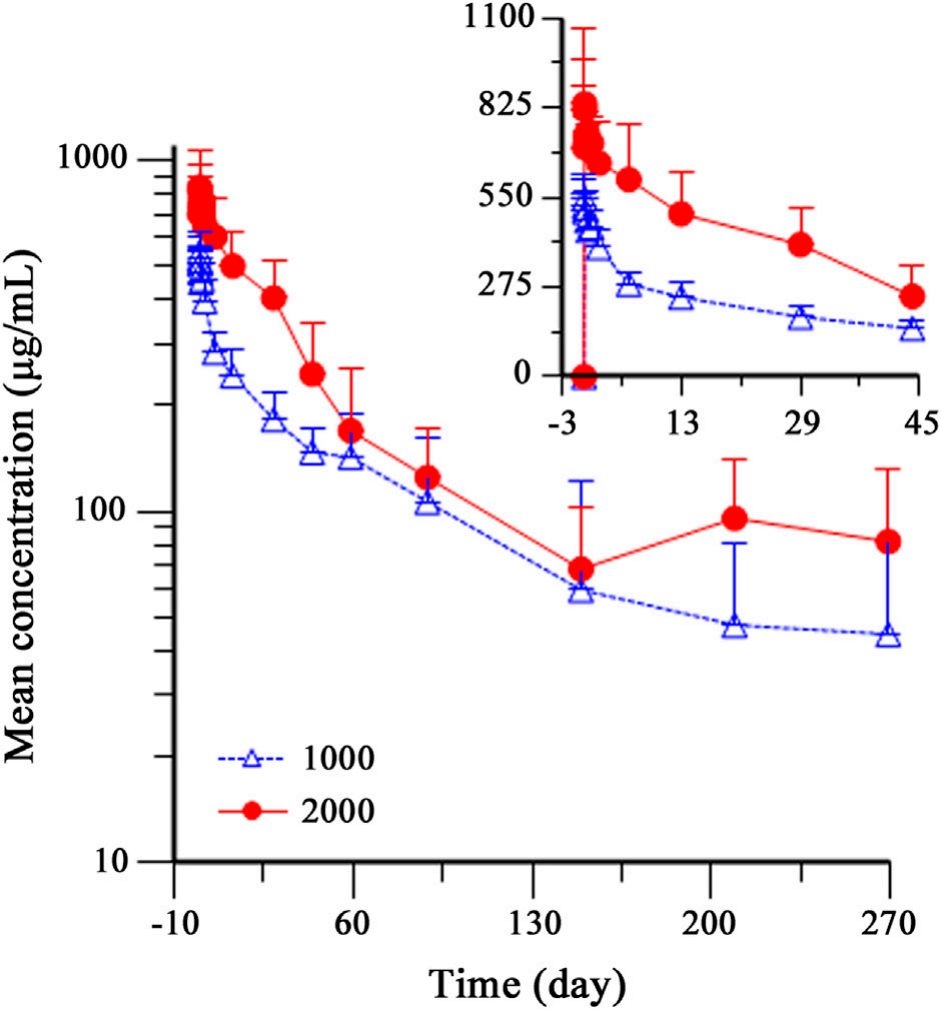
Mean (±SD) serum concentration-time profiles of HFB30132A in healthy Chinese subjects after single dose intravenous infusion on semi-logarithmic scale. Individual PK samples were collected on day 1 (dosing) and days 2, 3, 7, 14, 30, 45, 60, 90, 150, 210, and 270 post-dose, or at the time of early discontinuation. The profiles were plotted on a semi-log scale. The error bars represent the SD at each time point. SD, standard deviation.

As HFB30132A dose increased from 1,000 mg to 2,000 mg, the overall exposures increased accordingly. Figure 2 shows the serum concentration-time profiles of HFB30132A after single dose IV infusion on semi-logarithmic scale. The mean C_max_ and AUCs values increased as the dose increased from 1,000 to 2000 mg. The mean C_max_ value was 570.18 μg/mL in 1,000 mg group and 898.65 μg/mL in 2,000 mg group. The mean AUC_0-t_ and AUC_0-∞_ values were 644,749.42 h*μg/mL and 806,127.47 h*μg/mL in 1,000 mg group, and increased to 1,046,209.06 h*μg/mL and 1,299,190.74 h*μg/mL in 2,000 mg group. The median T_max_ was 2.5 h in either 1,000 mg or 2,000 mg group. The highest T_max_ was 5.5 h in 1,000 mg group, and 144 h in 2,000 mg group. From day 45 to the end of the study, the mean serum concentration of HFB30132A decreased slowly and maintained quantifiable in both dose groups. The estimated PK parameters were similar between the two dose groups. The mean t_½_, Vd, and CL were 107.09 days, 4,924.82 mL, and 1.38 mL/h in 1,000 mg group, and 89.36 days, 4,818.59 mL, and 1.59 mL/h in 2000 mg group, respectively. The PK parameters of HFB30132A are summarized in Table 4.

**TABLE 4:** PK parameters of HFB30132A after single dose intravenous infusion in healthy Chinese subjects.

PK parameter	HFB30132A 1000 mg (N = 8)	HFB30132A 2000 mg (N = 8)
C_max_ (μg/mL)	570.18 (75.69)	898.65 (207.24)
T_max_ (h)	2.50 (1.5–5.5)	2.50 (1.5–144)
AUC_0-t_ (h*μg/mL)	644,749.42 (228,802.56)	1,046,209.06 (205,066.94)
AUC_0-∞_ (h*μg/mL)	806,127.47 (308,537.61)	1,299,190.74 (236,157.93)
CL (mL/h)	1.38 (0.42)	1.59 (0.30)
Vd (mL)	4,924.82 (3,686.00)	4,818.59 (1,250.17)
t_1/2_ (h)	2570.11 (1885.09)	2144.75 (634.18)

Data are presented as mean (SD) except T_max_, which is expressed as median (minimum, maximum). PK, pharmacokinetic; N, number of subjects; C_max_, maximum concentration; T_max_, time to reach maximum concentration; AUC_0-t_, area under the serum concentration-time curve from time 0 to the time of the last quantifiable concentration; AUC_0-∞_, area under the curve from time 0 to infinity; CL, clearance; Vd, volume of distribution; t_1/2_, elimination half-life.

### 3.4 PPK modeling

The final PPK model included the following covariate-parameter relations: BILI on V1 (central compartment); WT and MONO on V2 (peripheral compartment 1); BILI on Q2 (inter-compartment clearance between V1 and V2); RBC and HGB on Q3 (inter-compartment clearance between V1 and V3). The parameter estimates of the final model were listed in Table 5. The equations for calculating the final model parameters were shown below:

$$CL=0.015\;\left(L/h\right)$$

$$V1=2.3\times {\left(\frac{BILI}{7.85}\right)}^{0.3}\times e^{\eta_{V1}}\;\left(L\right)$$

$$Q2=0.005\times {\left(\frac{BILI}{7.85}\right)}^{-1.2}\;\left(L/h\right)$$

$$V2=1.5\times {\left(\frac{WT}{64.58}\right)}^{6}\times {\left(\frac{MONO}{0.34}\right)}^{-2.2}\times e^{\eta_{V2}}\;\left(L\right)$$

$$Q3=0.0022\times {\left(\frac{RBC}{4.73}\right)}^{-4.2}\times {\left(\frac{HGB}{147.5}\right)}^{4.3}\;\left(L/h\right)$$

$$V3=33\times e^{\eta_{V3}}$$

**TABLE 5:** Final parameter estimates for the population pharmacokinetic model of HFB30132A.

Final model					
Parameter	Estimate	SE	RSE (%)	CV%	Shrinkage (%)
CL	0.0151	7.24	47,947.02	-	-
V1	2.33	103.00	4,420.60	-	-
Q2	0.00	0.36	7,228.92	-	-
V2	1.47	34.80	2367.35	-	-
Q3	0.0022	0.0030	134.84	-	-
V3	33.10	75.80	229.00	-	-
Q2_BILI1_	−1.18	0.68	57.63	-	-
Q3_HGB1_	4.30	1.88	43.72	-	-
Q3_RBC1_	−4.16	0.63	15.24	-	-
V1_BILI1_	0.30	1.45	480.13	-	-
V2_MONO1_	−2.20	6.08	276.36	-	-
V2_WEIGHTBL1_	6.00	0.00029	0.0049	-	-
η_V1_IIV_	0.0102	0.0024	23.43	10.10	14.50
η_V2_IIV_	0.0529	0.02	45.18	23.00	51.80
η_V3_IIV_	1.10	0.35	32.18	104.88	51.20
σ_Prop_	0.0413	0.00049	1.18	-	5.10

SE, standard error; RSE, relative standard error; CV, coefficient of variation; CL, clearance; V1, central compartment; V2, peripheral compartment 1; Q2, inter-compartment clearance between central compartment and peripheral compartment 1; V3, peripheral compartment 1; Q3, inter-compartment clearance between central compartment and peripheral compartment 2; BILI, bilirubin; HGB, hemoglobin; RBC, red blood count; MONO, monocyte; IIV, interindividual variability.

**FIGURE 3:**
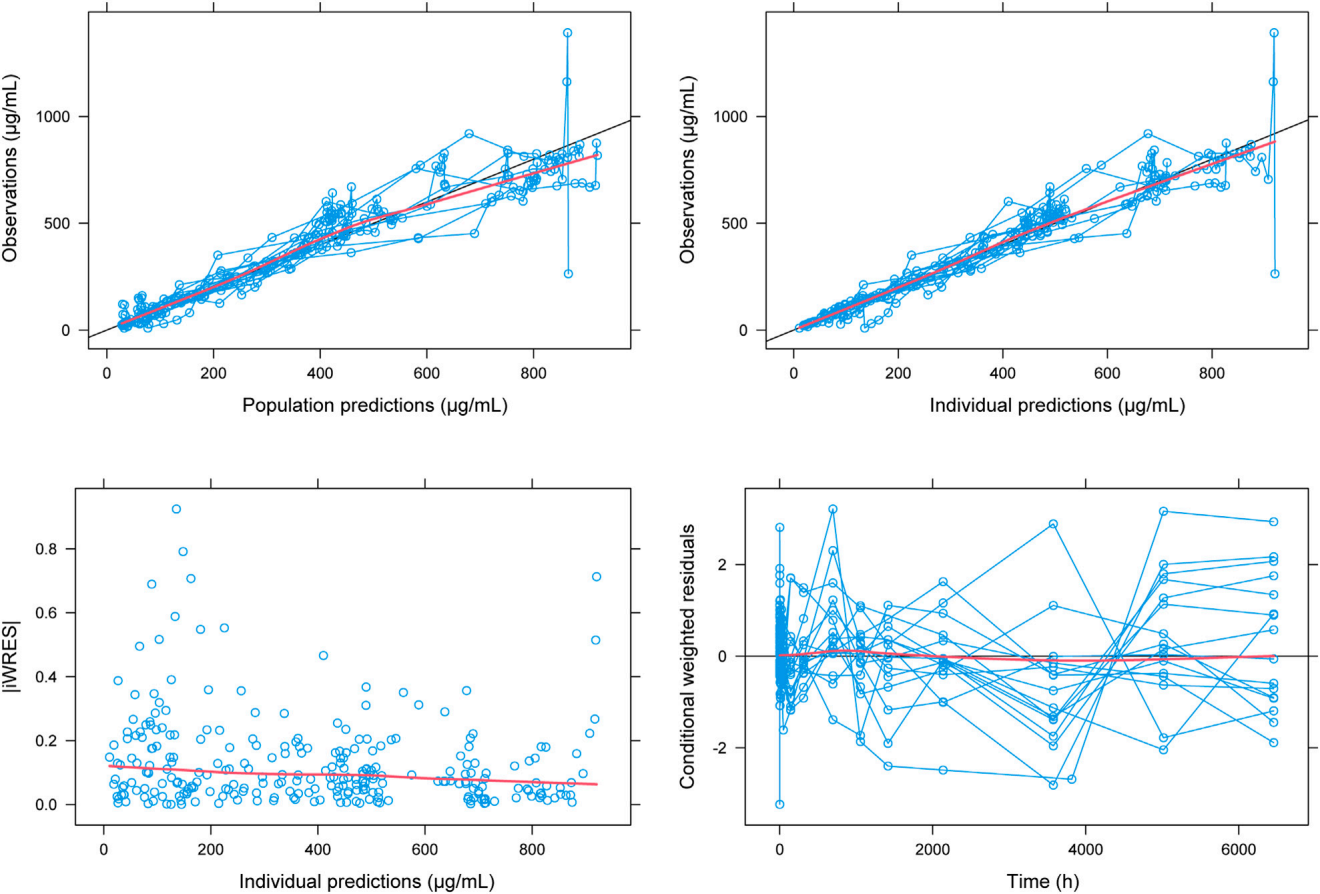
Fit plots for population pharmacokinetic model of HFB30132A. The blue line indicates the unit line. The red line indicates the locally weighted regression line (Loess).

The goodness of fit plots and individual fitting graph for PPK model of HFB30132A are provided in Figure 3. The predicted concentrations were approximate to the observed data. The distribution of the plots was nearly symmetrical across the unit line.

**FIGURE 4:**
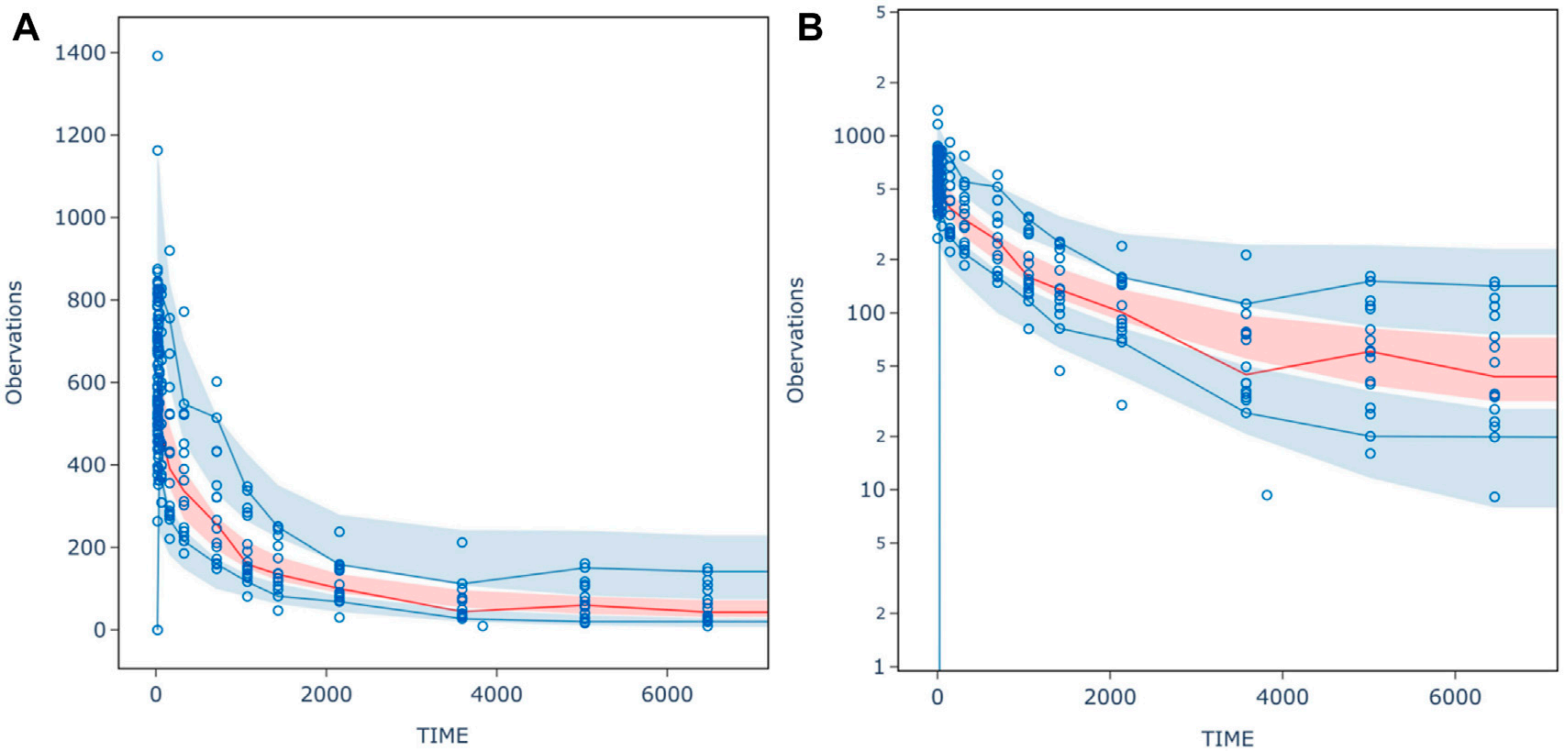
**(A)** normal scale **(B)** semi- logarithm scale prediction-corrected visual predictive check (pcVPC) for inal population PK model of HFB30132A. The circles indicate the observed concentration corrected by prediction. The red solid line is the median for prediction-corrected observed data, and the deep shaded area was 95% confidence interval. The blue solid lines represent 90% and 10% percentiles for prediction-corrected observed data, respectively, whereas the shallow shaded area is the corresponding 95% confidence interval.

The VPC result for PPK model was shown in Figure 4. The median of observed data (solid line) was almost within the 95% confidence interval (CI) of the simulated data (red shaded area). Similarly, the 90% and 10% percentile of the observed data were also within 95% CI of the simulated data (blue shaded area). The final PPK model characterized the PK profiles of HFB30132A quite well.

### 3.5 Immunogenicity

ADA test did not detect any anti-HFB30132A antibodies in the serum of any subject during serial testing. A single dose IV infusion of HFB30132A did not induce immunogenic response in this study.

## 4 Discussion

This phase 1 study demonstrated the tolerable and safe profile of HFB30132A, an anti-SARS-CoV-2 mAb, in healthy Chinese adults following single dose IV infusion. HFB30132A is derived from a convalescent COVID-19 patient and specifically binds to SARS-CoV-2 S protein RBD. It was engineered as an IgG4 antibody containing 4 mutations. The YTE mutation in the IgG4 Fc moiety could reduce the binding to FcγR, and so decrease the risk of ADE effect (Liu et al., 2019; Graham, 2020), and may increase the binding to FcRn, and so enable a longer half-life, and increased distribution to the respiratory system mucosa in comparison with IgG1 antibodies (Dall'Acqua et al., 2006; Guo et al., 2021; Tabrizi et al., 2019; Spiess et al., 2013).

Only 5 cases of laboratory abnormalities in 4 subjects were considered as ADRs, which were Grade 1 or 2 in severity. No other clinically significant abnormal findings were reported in any subject. The ADRs were not considered as safety signal. They were probably due to interindividual variability based on the temporality, actual values, and lack of symptoms or clinical relevance.

After a single dose IV infusion of HFB30132A 1,000 mg or 2,000 mg, the median T_max_ was 2.5 h in both dose groups. The mean serum concentration of HFB30132A decreased slowly and maintained quantifiable in both dose groups from day 45 to the end of the study. The mean t_½_ value was 89.36 days in 2,000 mg group and 107.09 days in 1,000 mg group. The slight difference between groups might be attributable to FcRn saturation, and the excessive mAb could be metabolized, resulted in shorter t_1/2_ in the high-dose group. Accordingly, the exposure PK parameters (C_max_, AUC_0-t_ and AUC_0-∞_) were also slightly less than proportional. The PK profiles of HFB30132A in healthy Chinese adults indicate dose dependency and an extended half-life.

The covariates WT, RBC, HGB, MONO, and BILI were finally adopted to develop the PPK model. The inclusion of the relations of RBC, HGB and MONO on PK parameters was difficult to explain from PK perspective. However, since the IgG4 inhibitors (HFB30132A is also an IgG4 inhibitor) could affect RBC and platelet, the pharmacodynamic effect of HFB30132A might account for the relationship between PK parameters and RBC/HGB/MONO (Velliquette et al., 2019).

During the process of PPK modeling, the performance of base model was evaluated using two-compartment model, three-compartment model, and target-mediated drug disposition (TMDD) model. Three-compartment model resulted in a significantly lower OFV than two-compartment model (2204.0 vs. 2408.4). The OBJ for the TMDD model was lower than that for two-compartment model (2375.7), but it was still higher than that for three-compartment model. These results indicate that HFB30132A undergoes a linear elimination or healthy Chinese volunteers lack the specific targets which mediate the disposition of HFB30132A. Therefore, a three-compartment model was selected as the base model.

ADA test did not report positive result in any subject, suggesting low immunogenicity after single dose infusion of HFB30132A. HFB30132A was well tolerated and safe with a long half-life in Chinese healthy volunteers. The serum samples collected until day 90 were used to test the titers of neutralizing antibodies against multiple SARS-CoV-2 variants using a pseudovirus neutralization assay. The results showed that HFB30132A maintained high level of neutralizing activity against SARS-CoV-2 wild-type and the Alpha, Delta, Lambda, Kappa, Iota, and Mu variants up to 90 days, but was inactive against the Omicron BA.2 variant (manuscript under review).

A total number of 20 participants is too small to conclude on the clinical value of HFB30132A, but its Fc moiety enabling long half-life provides the base for further study to clarify its real utility in pre-exposure prophylaxis of COVID-19. As the predominant Omicron BA.2 variant spreads globally, the FDA issued an EUA for emergency use of Evusheld (tixagevimab co-packaged with cilgavimab) for pre-exposure prophylaxis of COVID-19 in adults and children (12 years of age and older weighing at least 40 kg) (National Institutes of Health, 2022). More efficacy and safety data of single mAb such as HFB30132A or cocktails of mAbs are required for prophylaxis and treatment of COVID-19.

## 5 Conclusion

The safety, tolerability, pharmacokinetics, and immunogenicity of HFB30132A, a new anti-SARS-CoV-2 mAb, were evaluated in a phase 1, randomized, double-blind, placebo-controlled study in healthy Chinese adults. After single dose IV infusion of 1,000 mg and 2000 mg, HFB30132A showed good safety profile and an extended half-life in Chinese healthy adults. All subjects tolerated the treatment quite well. HFB30132A did not induce anti-HFB30132A antibody. The long half-life of HFB30132A may provide longer time of protection. These preliminary findings will be combined with the *in vitro* anti-SARS-CoV-2 activities to propose an optimal dosing regimen for use in future clinical trials to test the real utility of HFB30132A in prevention of COVID-19.

## Data Availability

The original contributions presented in the study are included in the article/Supplementary Material, further inquiries can be directed to the corresponding authors.
